# Supplementary material for: Device-assessed sleep and physical activity in individuals recovering from a hospital admission for COVID-19: a multicentre study
Source: Int J Behav Nutr Phys Act. 2022 Jul 28;19:94. doi: 10.1186/s12966-022-01333-w (PMC9330990; doi:10.1186/s12966-022-01333-w)
Supplement: Supplementary file 2 — Additional file 2. Supplementary material. [file 12966_2022_1333_MOESM2_ESM.docx]

**Supplementary material**

[Supplementary methods 2](#_Toc93337549)

[Accelerometer data processing 2](#_Toc93337550)

[Definitions of physical activity and sleep variables 2](#_Toc93337551)

[Disease definitions 3](#_Toc93337552)

[Covariates 4](#_Toc93337553)

[Comparative cohorts 4](#_Toc93337554)

[Supplementary results 6](#_Toc93337555)

[Supplementary tables 6](#_Toc93337556)

[Supplementary figures 15](#_Toc93337557)

[References 18](#_Toc93337558)

# Supplementary methods

## Accelerometer data processing

Data were downloaded using GENEActiv PC software V.3.2 and accelerometer files were processed with R-package GGIR version 2.2-0 (<http://cran.r-project.org>) [1].

Sleep characteristics were obtained using a validated automated sleep detection algorithm [2]. This algorithm facilitates detection of the sleep period time window (SPT-window) without the use of sleep diaries. The SPT-window refers to the time window starting at sleep onset and ending when waking up after the last sleep episode of the night.

Individual nights were excluded if the monitor was not worn on a night, the sleep window was <3 h, >12 h, or the number of sleep episodes per night was ≤5 or ≥30 as in previous research [3, 4]. In addition, nights with sleep efficiency <50% were visually inspected and unrealistic nights removed (e.g., sleep window clearly erroneously detected). Out of 13 348 nights, 1 452 (~11%) individual nights were excluded based on these criteria. Over a third of these were excluded due to visual inspection showing the monitor not being worn on an individual night. Following exclusions, the median number of valid nights per participant available for analyses was 14 [IQR 12 – 14 nights].

## Definitions of physical activity and sleep variables

| ***Daily physical activity variables*** | **Definition** |
| --- | --- |
| Average acceleration (m*g)* | A proxy for physical activity volume |
| Moderate-to-vigorous intensity physical activity (minutes) | Accumulated in ≥1-minute bouts (>100 m*g*) [5] |
| Light-intensity activity (minutes) | Time accumulated with an acceleration between 40 and 100 m*g* [6] |
| Inactive time (hours) | Time accumulated during the waking day below 40 m*g* [6] |
| Intensity of the most active continuous 30 and 10 minutes/day (m*g*) | Acceleration value corresponding to the 25th percentile of the distribution during the most active period, therefore 75% of the time within these bouts is spent above this value |
| ***Weekly physical activity characteristics*** |  |
| Number of days per week with 10- and 30-minute continuous MVPA | Weekly variables were limited to participants with at least seven valid days of data. A continuous 10 (or 30) minute moderate-to-vigorous activity session is evident when the intensity corresponding with the 25th percentile of the distribution for the 10 (or 30) continuous minutes is >100 m*g* (e.g., walking), i.e., there is an allowance for up to 25% of time to drop below the threshold consistent with standard bout definitions (e.g., Troiano et al., 2008) [7] |
| ***Sleep variables*** |  |
| Total sleep time (hours) | Total accumulated sleep within the sleep window |
| Sleep efficiency (%) | The ratio of total sleep time to the duration of the sleep window |
| Mid-sleep variability (SD in minutes) | Within-person standard deviation of mid-sleep time (mid-point between sleep onset and waking time). Sleep mid-point variability describes how variable people are in the timing of their sleep window. |

## Disease definitions

WHO clinical progression scale [8], defined as:

1. Class 3-4 = no continuous supplemental oxygen needed,
2. Class 5 = continuous supplemental oxygen only,
3. Class 6 = continuous positive airway pressure ventilation (CPAP), bi-level positive airway pressure (BIPAP) or high flow nasal oxygen,
4. Class 7-9 = invasive mechanical ventilation (IMV) or extra-corporeal membrane oxygenation (ECMO).

Four recovery clusters from a battery of patient reported outcomes and physical tests [9]:

- 1. Very severe mental and physical health impairment,
  2. Severe mental and physical health impairment,
  3. Moderate mental and physical health impairment with pronounced cognitive impairment,
  4. Mild mental and physical health impairment

The battery of patient reported outcomes and physical tests used to create these clusters, which were assessed using validated tools, included:

| **Symptom** | **Method** |
| --- | --- |
| Breathlessness | Dyspnoea-12 [10] |
| Fatigue | FACIT (Functional Assessment of Chronic Illness Therapy) [11] |
| Anxiety | GAD-7 (General Anxiety Disorder 7 Questionnaire) [12] |
| Depression | PHQ-9 (Patient Health Questionnaire – 9) [13] |
| Post-traumatic stress disorder | PCL-5 (Post Traumatic Stress Disorder Checklist) [14] |
| Physical performance | SPPB (Short Physical Performance Battery) [15] |
| Cognition | MoCA (Montreal Cognitive Assessment) [16] |

## Covariates

| **Covariate** | **Method** |
| --- | --- |
| Ethnicity | Ethnicity was recorded according to census definitions and classified as White (English, Welsh, Scottish, Northern Irish or British, Irish, Gypsy or Irish Traveller any other White background), Black (African, Caribbean, any other Black, African or Caribbean background), South Asian (Indian, Pakistani, Bangladeshi) or other (including mixed) ethnicities. |
| Comorbidity | Derived from a comprehensive medical history taken during the hospital admission and categorised as none, one or two or more comorbidities. |
| Index of Multiple Deprivation | Based on seven domains of deprivation: income, employment, education, skills and training, health and disability, crime, barriers to housing services, living environment and centred on participants postcodes and categorised in quintiles based on nationally derived thresholds. |

## Comparative cohorts

The SMART Work and Life (SWL) study recruited adult office workers aged ≥18 years within local Councils in the Leicester, Manchester and Liverpool areas between 2018 and 2019. Details of the study have been published [17]. Participants wore an Axivity AX3 (Axivity Ltd, Newcastle, UK) accelerometer their non-dominant wrist 24 h/day for 7 days, with data recorded at 100 Hz. Previous studies showed that physical activity and sleep outcomes from the GENEActiv and Axivity accelerometers are comparable when generated using methods as described above [18, 19]. When worn on the non-dominant wrist, direct measures of acceleration, outcomes based on the pattern of accelerations, inactive time and MVPA outcomes could be considered equivalent between the GENEActiv and Axivity accelerometers [18]. Similarly, sleep estimates such total sleep time and sleep efficiency were equivalent between these brands when worn on the non-dominant wrist [19]. Because the SWL cohort was younger, to match with the PHOSP sample, age was split into tertiles and only participants in the upper tertile were included in the analysis (***Supplementary Table S5***).

The CODEC study (Chronotype of Patients with Type 2 Diabetes and Effect on Glycaemic Control) recruited people with type 2 diabetes throughout the East Midlands between 2017 and 2019. The CODEC cohort study has been described in detail previously [20, 21]. Individuals wore the same accelerometer as in PHOSP-COVID (GENEActiv) for 7 days, with data recorded at 100 Hz. Characteristics of the CODEC sample, including the number included, were similar to the PHOSP sample (***Supplementary Table S5***).

# Supplementary results

## Supplementary tables

**Supplementary Table S1: Participant characteristics by missing accelerometer data status.**

|  |  | **Missing** | | **Complete** | |
| --- | --- | --- | --- | --- | --- |
| Variable |  | **Count** | **Column %** | **Count** | **Column %** |
| WHO disease severity class* | Class 3-4 | 87 | 24.0% | 139 | 19.4% |
|  | Class 5 | 123 | 34.0% | 255 | 35.7% |
|  | Class 6 | 69 | 19.1% | 116 | 16.2% |
|  | Class 7-9 | 83 | 22.9% | 205 | 28.7% |
| Recovery cluster | Cluster 1: Very severe | 45 | 12.4% | 86 | 12.0% |
|  | Cluster 2: Severe | 51 | 14.1% | 108 | 15.1% |
|  | Cluster 3: Moderate | 38 | 10.5% | 89 | 12.4% |
|  | Cluster 4: Mild | 112 | 30.9% | 238 | 33.3% |
|  | Missing | 116 | 32.0% | 194 | 27.1% |
| Sex | Female | 131 | 36.2% | 253 | 35.4% |
|  | Male | 231 | 63.8% | 462 | 64.6% |
| Ethnicity | White | 211 | 58.3% | 499 | 69.8% |
|  | South Asian | 74 | 20.4% | 92 | 12.9% |
|  | Black | 40 | 11.0% | 49 | 6.9% |
|  | Mixed | 8 | 2.2% | 15 | 2.1% |
|  | Other | 19 | 5.2% | 28 | 3.9% |
|  | Missing | 10 | 2.8% | 32 | 4.5% |
| IMD Quintile** | 1 - most deprived | 80 | 22.1% | 135 | 18.9% |
|  | 2 | 76 | 21.0% | 166 | 23.2% |
|  | 3 | 75 | 20.7% | 127 | 17.8% |
|  | 4 | 55 | 15.2% | 141 | 19.7% |
|  | 5 - least deprived | 53 | 14.6% | 145 | 20.3% |
|  | Missing | 23 | 6.4% | 1 | 0.1% |
| **Continuous Variables** | | Mean | SD | Mean | SD |
| Age (years) | | 55 | 13 | 59 | 13 |
| BMI*** | | 32.1 | 7.5 | 31.0 | 6.8 |

*WHO clinical progression scale: 3-4 = no continuous supplemental oxygen needed, 5 = continuous supplemental oxygen only, 6 = continuous positive airway pressure ventilation (CPAP), bi-level positive airway pressure (BIPAP) or high flow nasal oxygen, 7-9 = invasive mechanical ventilation (IMV) or extra-corporeal membrane oxygenation (ECMO). ** IMD = Index of Multiple Deprivation; ***BMI = body mass index

**Supplementary Table S2. Sleep variables across WHO classes of acute disease severity and four recovery clusters excluding healthcare workers (N = 518).**

| **WHO classes** | | | | | | | | | | |
| --- | --- | --- | --- | --- | --- | --- | --- | --- | --- | --- |
|  | **Women** | | | | **Men** | | | |  |  |
| **Sleep variables** | **Class 3-4** | **Class 5** | **Class 6** | **Class 7-9** | **Class 3-4** | **Class 5** | **Class 6** | **Class 7-9** | **P for class** | **P for class x sex** |
| Total sleep time (hrs/night) | 6.9 (6.6, 7.3) | 7.0 (6.7, 7.4) | 7.01 (6.59, 7.60) | 6.8 (6.3, 7.2) | 6.6 (6.3, 7.0) | 6.7 (6.4, 7.0) | 6.7 (6.3, 7.0) | 6.6 (6.3, 6.9) | 0.723 | 0.878 |
| Sleep efficiency (%) | 87.1 (85.1, 89.0) | 87.1 (85.3, 88.9) | 86.56 (84.05, 89.06) | 86.2 (83.9, 88.5) | 86.6 (84.8, 88.5) | 85.8 (84.3, 87.3) | 86.0 (84.1, 87.8) | 86.3 (84.7, 87.8) | 0.854 | 0.799 |
| Sleep midpoint variability (minutes) | 91.4 (62.1, 120.6) | 87.4 (60.8, 113.9) | 74.72 (37.04, 112.39) | 115.8 (81.2, 150.4) | 100.6 (72.8, 128.4) | 86.6 (64.1, 109.0) | 107.1 (79.1, 135.1) | 85.7 (62.1, 109.4) | 0.616 | 0.103 |
| **Recovery clusters** | | | | | | | | | | |
|  | **Women** | | | | **Men** | | | |  |  |
| **Sleep variables** | **Cluster 4: Mild** | **Cluster 3: Moderate** | **Cluster 2: Severe** | **Cluster 1: Very Severe** | **Cluster 4: Mild** | **Cluster 3: Moderate** | **Cluster 2: Severe** | **Cluster 1: Very Severe** | **P for class** | **P for class x sex** |
| Total sleep time (hrs/night) | 7.2 (6.8, 7.6) | 6.9 (6.4, 7.4) | 7.1 (6.7, 7.6) | 6.7 (6.2, 7.2) | 6.7 (6.4, 7.0) | 6.5 (6.1, 6.9) | 7.1 (6.7, 7.5) | 6.8 (6.3, 7.2) | 0.144 | 0.356 |
| Sleep efficiency (%) | 86.9 (84.8, 88.9) | 87.1 (84.5, 89.6) | 87.2 (84.9, 89.54) | 86.3 (83.7, 89.0) | 86.2 (84.6, 87.8) | 85.3 (83.4, 87.3) | 86.0 (84.3, 88.5) | 85.4 (83.2, 87.7) | 0.808 | 0.934 |
| Sleep midpoint variability (minutes) | 87.4 (56.9, 117.9) | 88.9 (50.3, 127.4) | 78.3 (43.1, 113. 5) | 100.0 (60.7, 139.3) | 85.9 (62.1, 109.7) | 98.4 (69.3, 127.5) | 74.1 (42.9, 105.3) | 116.7 (83.1, 150.2) | 0.172 | 0.874 |

Data reported as marginal mean (95% CI). Adjusted for age, sex, ethnicity, deprivation, number of comorbidities, season of data collection, and number of wear nights (sleep variables). Models included 518 participants with complete cluster and covariate data.

**Supplementary Table S3: Associations of independent cluster variables with physical activity and sleep variables.**

| **Exposure** | **Outcome** | b (95% CI) | P for main effect | P for interaction by sex |
| --- | --- | --- | --- | --- |
| **Breathlessness (Dyspnoea-12)** | **Physical activity variables** |  |  |  |
|  | Physical activity volume (m*g*) | -0.75 (-1.25, -0.25) | 0.003 | 0.867 |
|  | Moderate to vigorous intensity physical activity (minutes/day) | -2.64 (-4.08, -1.20) | 0.001 | 0.351 |
|  | Light intensity physical activity (minutes/day) | -6.03 (-9.93, -2.14) | 0.002 | 0.380 |
|  | Inactivity (minutes/day) | 4.41 (-2.95, 11.77) | 0.240 | 0.653 |
|  | Intensity of the most active continuous 30 minutes (m*g*) | -4.37 (-7.6, -1.14) | 0.008 | 0.683 |
|  | Intensity of the most active continuous 10 minutes (m*g*) | -7.20 (-11.61, -2.79) | 0.001 | 0.573 |
|  | **Sleep variables** |  |  |  |
|  | Total sleep time (hrs/night) | 0.04 (-0.05, 0.12) | 0.405 | 0.585 |
|  | Sleep efficiency (%) | -0.23 (-0.65, 0.19) | 0.283 | 0.662 |
|  | Sleep midpoint variability (minutes) | 8.52 (2.12, 14.93) | 0.009 | 0.888 |
| **Fatigue (FACIT)** | **Physical activity variables** |  |  |  |
|  | Physical activity volume (m*g*) | -1.26 (-1.79, -0.72) | 0.001 | 0.762 |
|  | Moderate to vigorous intensity physical activity (minutes/day) | -4.15 (-5.67, -2.64) | 0.001 | 0.112 |
|  | Light intensity physical activity (minutes/day) | -8.94 (-13.07, -4.81) | 0.001 | 0.763 |
|  | Inactivity (minutes/day) | 10.47 (2.61, 18.33) | 0.009 | 0.750 |
|  | Intensity of the most active continuous 30 minutes (m*g*) | -7.29 (-10.63, -3.94) | 0.001 | 0.175 |
|  | Intensity of the most active continuous 10 minutes (m*g*) | -11.32 (-15.88, -6.75) | 0.001 | 0.099 |
|  | **Sleep variables** |  |  |  |
|  | Total sleep time (hrs/night) | 0.02 (-0.06, 0.11) | 0.594 | 0.661 |
|  | Sleep efficiency (%) | -0.05 (-0.50, 0.40) | 0.834 | 0.805 |
|  | Sleep midpoint variability (minutes) | 11.58 (4.75, 18.41) | 0.001 | 0.409 |

Data reported as beta-coefficients (95% CI). Adjusted for age, sex, ethnicity, deprivation, number of comorbidities, season of data collection, and number of wear days (physical activity variables) or wear nights (sleep variables). FACIT = Functional Assessment of Chronic Illness Therapy.

| **Exposure** | **Outcome** | b (95% CI) | P for main effect | P for interaction by sex |
| --- | --- | --- | --- | --- |
| **Physical performance (SPPB)** | **Physical activity variables** |  |  |  |
|  | Physical activity volume (m*g*) | 0.90 (0.34, 1.47) | 0.002 | 0.058 |
|  | Moderate to vigorous intensity physical activity (minutes/day) | 3.65 (2.00, 5.29) | 0.001 | 0.037 |
|  | Light intensity physical activity (minutes/day) | 5.59 (1.33, 9.85) | 0.010 | 0.164 |
|  | Inactivity (minutes/day) | -15.06 (-23.17, -6.95) | 0.001 | 0.426 |
|  | Intensity of the most active continuous 30 minutes (m*g*) | 7.21 (3.67, 10.76) | 0.001 | 0.016 |
|  | Intensity of the most active continuous 10 minutes (m*g*) | 9.52 (4.64, 14.40) | 0.001 | 0.034 |
|  | **Sleep variables** |  |  |  |
|  | Total sleep time (hrs/night) | 0.01 (-0.09, 0.09) | 0.970 | 0.708 |
|  | Sleep efficiency (%) | -0.12 (-0.59, 0.34) | 0.604 | 0.530 |
|  | Sleep midpoint variability (minutes) | -2.74 (-9.94, 4.47) | 0.457 | 0.055 |
| **Cognition (MoCA)** | **Physical activity variables** |  |  |  |
|  | Physical activity volume (m*g*) | -0.03 (-0.60, 0.54) | 0.922 | 0.554 |
|  | Moderate to vigorous intensity physical activity (minutes/day) | 0.98 (-0.72, 2.68) | 0.258 | 0.983 |
|  | Light intensity physical activity (minutes/day) | -2.52 (-6.84, 1.80) | 0.253 | 0.600 |
|  | Inactivity (minutes/day) | 3.08 (-5.19, 11.35) | 0.466 | 0.953 |
|  | Intensity of the most active continuous 30 minutes (m*g*) | 2.12 (-1.68, 5.91) | 0.274 | 0.637 |
|  | Intensity of the most active continuous 10 minutes (m*g*) | 4.28 (-0.92, 9.48) | 0.107 | 0.742 |
|  | **Sleep variables** |  |  |  |
|  | Total sleep time (hrs/night) | 0.06 (-0.03, 0.16) | 0.180 | 0.681 |
|  | Sleep efficiency (%) | 0.40 (-0.07, 0.87) | 0.092 | 0.371 |
|  | Sleep midpoint variability (minutes) | -6.92 (-14.13, 0.29) | 0.060 | 0.467 |

Data reported as beta-coefficients (95% CI). Adjusted for age, sex, ethnicity, deprivation, number of comorbidities, season of data collection, and number of wear days (physical activity variables) or wear nights (sleep variables). SPPB = short physical performance battery, MoCA = Montreal Cognitive Assessment.

| **Exposure** | **Outcome** | b (95% CI) | P for main effect | P for interaction by sex |
| --- | --- | --- | --- | --- |
| **Anxiety (GAD-7)** | **Physical activity variables** |  |  |  |
|  | Physical activity volume (m*g*) | -0.30 (-0.82, 0.22) | 0.256 | 0.114 |
|  | Moderate to vigorous intensity physical activity (minutes/day) | -1.57 (-3.06, -0.09) | 0.038 | 0.832 |
|  | Light intensity physical activity (minutes/day) | -3.52 (-7.56, 0.52) | 0.088 | 0.314 |
|  | Inactivity (minutes/day) | 0.19 (-7.46, 7.84) | 0.961 | 0.900 |
|  | Intensity of the most active continuous 30 minutes (m*g*) | -2.56 (-5.84, 0.72) | 0.126 | 0.804 |
|  | Intensity of the most active continuous 10 minutes (m*g*) | -4.35 (-8.82, 0.11) | 0.056 | 0.772 |
|  | **Sleep variables** |  |  |  |
|  | Total sleep time (hrs/night) | 0.02 (-0.07, 0.11) | 0.645 | 0.854 |
|  | Sleep efficiency (%) | -0.37 (-0.81, 0.06) | 0.093 | 0.984 |
|  | Sleep midpoint variability (minutes) | 9.45 (2.76, 16.15) | 0.006 | 0.327 |
| **Depression (PHQ-9)** | **Physical activity variables** |  |  |  |
|  | Physical activity volume (m*g*) | -0.58 (-1.11, -0.05) | 0.031 | 0.341 |
|  | Moderate to vigorous intensity physical activity (minutes/day) | -2.35 (-3.86, -0.85) | 0.002 | 0.318 |
|  | Light intensity physical activity (minutes/day) | -5.91 (-9.99, -1.82) | 0.005 | 0.369 |
|  | Inactivity (minutes/day) | 4.34 (-3.41, 12.10) | 0.272 | 0.901 |
|  | Intensity of the most active continuous 30 minutes (m*g*) | -4.21 (-7.54, -0.88) | 0.013 | 0.791 |
|  | Intensity of the most active continuous 10 minutes (m*g*) | -7.01 (-11.55, -2.47) | 0.002 | 0.606 |
|  | **Sleep variables** |  |  |  |
|  | Total sleep time (hrs/night) | -0.02 (-0.10, 0.07) | 0.732 | 0.419 |
|  | Sleep efficiency (%) | -0.46 (-0.90, -0.02) | 0.039 | 0.774 |
|  | Sleep midpoint variability (minutes) | 10.35 (3.55, 17.15) | 0.003 | 0.762 |

Data reported as beta-coefficients (95% CI). Adjusted for age, sex, ethnicity, deprivation, number of comorbidities, season of data collection, and number of wear days (physical activity variables) or wear nights (sleep variables). GAD-7 = General Anxiety Disorder 7 Questionnaire, PHQ-9 = Patient Health Questionnaire – 9.

| **Exposure** | **Outcome** | b (95% CI) | P for main effect | P for interaction by sex |
| --- | --- | --- | --- | --- |
| **Post-traumatic stress disorder (PCL-5)** | **Physical activity variables** |  |  |  |
|  | Physical activity volume (m*g*) | -0.66 (-1.16, -0.15) | 0.011 | 0.575 |
|  | Moderate to vigorous intensity physical activity (minutes/day) | -2.53 (-3.98, -1.07) | 0.001 | 0.226 |
|  | Light intensity physical activity (minutes/day) | -5.35 (-9.28, -1.43) | 0.008 | 0.551 |
|  | Inactivity (minutes/day) | 3.68 (-3.8, 11.15) | 0.335 | 0.656 |
|  | Intensity of the most active continuous 30 minutes (m*g*) | -4.06 (-7.29, -0.83) | 0.014 | 0.768 |
|  | Intensity of the most active continuous 10 minutes (m*g*) | -6.56 (-10.98, -2.15) | 0.004 | 0.529 |
|  | **Sleep variables** |  |  |  |
|  | Total sleep time (hrs/night) | -0.01 (-0.1, 0.07) | 0.786 | 0.286 |
|  | Sleep efficiency (%) | -0.26 (-0.68, 0.17) | 0.242 | 0.387 |
|  | Sleep midpoint variability (minutes) | 12.57 (6.02, 19.11) | 0.001 | 0.237 |

Data reported as beta-coefficients (95% CI). Adjusted for age, sex, ethnicity, deprivation, number of comorbidities, season of data collection, and number of wear days (physical activity variables) or wear nights (sleep variables). PCL-5 = Post Traumatic Stress Disorder Checklist.

**Supplementary Table S4:** **Associations of physical performance with moderate to vigorous intensity physical activity and intensity of the most active continuous 30/10 minutes stratified by sex.**

|  |  | **Women** | | **Men** | |
| --- | --- | --- | --- | --- | --- |
| **Exposure** | **Outcome** | b (95% CI) | p for main effect | b (95% CI) | p for main effect |
| **Physical performance (SPPB)** |  |  |  |  |  |
|  | Moderate to vigorous intensity physical activity (minutes/day) | 2.11 (0.07, 4.14) | 0.042 | 4.51 (2.22, 6.81) | 0.001 |
|  | Intensity of the most active continuous 30 minutes (m*g*) | 2.92 (-0.65, 6.48) | 0.109 | 9.90 (4.79, 15.00) | 0.001 |
|  | Intensity of the most active continuous 10 minutes (m*g*) | 5.26 (-0.41, 10.92) | 0.069 | 12.04 (5.19, 18.90) | 0.001 |

Data reported as beta-coefficients (95% CI). Adjusted for age, ethnicity, deprivation, number of comorbidities, season of data collection, and number of wear days. SPPB = short physical performance battery.

**Supplementary Table S5: Participant characteristics in the CODEC (type 2 diabetes) and SWL (office workers) compared to the PHOSP-COVID cohorts.**

|  |  | **PHOSP** | | **CODEC** | | **SWL** | |
| --- | --- | --- | --- | --- | --- | --- | --- |
| **Categorical variables** |  | **Count** | **Column %** | **Count** | **Column %** | **Count** | **Column%** |
| Sex | Female | 253 | 35.4% | 236 | 34.5% | 165 | 71.1% |
|  | Male | 462 | 64.6% | 449 | 65.5% | 67 | 28.9% |
| Ethnicity | White | 499 | 69.8% | 576 | 84.1% | 175 | 75.4% |
|  | South Asian | 92 | 12.9% | 80 | 11.7% | 44 | 19.0% |
|  | Black | 49 | 6.9% | 21 | 3.1% | 5 | 2.2% |
|  | Other | 43 | 6.0% | 8 | 1.2% | 8 | 3.4% |
|  | Missing | 32 | 4.5% | 0 | 0.0% | 0 | 0.0% |
| IMD (quintile)* | 1 | 135 | 18.9% | 95 | 13.9% | 25 | 10.8% |
|  | 2 | 166 | 23.2% | 96 | 14.0% | 33 | 14.2% |
|  | 3 | 127 | 17.8% | 110 | 16.1% | 47 | 20.3% |
|  | 4 | 141 | 19.7% | 156 | 22.8% | 61 | 26.3% |
|  | 5 | 145 | 20.3% | 224 | 32.7% | 64 | 27.6% |
|  | Missing | 1 | 0.1% | 4 | 0.6% | 2 | 0.9% |
| Comorbidities | No comorbidity | 205 | 28.7% | 95 | 13.9% | 178 | 76.7% |
|  | 1 comorbidity | 145 | 20.3% | 186 | 27.2% | 47 | 20.3% |
|  | 2+ comorbidities | 365 | 51.0% | 404 | 59.0% | 7 | 3.0% |
| Continuous variables | | Mean | SD | Mean | SD | Mean | SD |
| Age (years) | | 59 | 13 | 64 | 8 | 55.8 | 3.4 |
| BMI (kg/m^2^)** | | 31 | 6.8 | 30.9 | 5 | 26.9 | 5.5 |

* IMD = Index of Multiple Deprivation; **BMI = body mass index

**Supplementary Table S6: Physical activity and sleep variables in the CODEC (type 2 diabetes) and SWL (office workers) compared to the PHOSP-COVID cohorts.**

|  | **PHOSP (n = 715)** | | **CODEC (n = 685)** | | **SWL (n = 236)** | | **P for group** | **P for group x sex** |
| --- | --- | --- | --- | --- | --- | --- | --- | --- |
|  | **Women** | **Men** | **Women** | **Men** | **Women** | **Men** |  |  |
| **Physical activity variables** |  |  |  |  |  |  |  |  |
| Physical activity volume (m*g*) | 20.9  (19.5, 22.2) | 22.0  (20.8, 23.2) | 22.2  (20.9, 23.5) | 22.9  (21.8, 24.1) | 23.3  (22.0, 24.7) | 26.0  (24.2, 27.8) | <0.001 | 0.218 |
| Moderate to vigorous intensity physical activity (minutes/day) | 17.7  (13.3, 22.0) | 25.9  (22.0, 29.9) | 19.7  (15.6, 23.9) | 26.1  (22.4, 29.9) | 30.9  (26.5, 35.3) | 44.7  (38.7, 50.7) | <0.001 | 0.118 |
| Light intensity physical activity (minutes/day) | 168.5  (157.8, 179.2) | 158.7  (148.8, 168.5) | 184.3  (174.3, 194.3) | 177.0  (167.8, 186.1) | 176.0  (165.5, 186.5) | 156.2  (141.9, 170.6) | 0.004 | 0.340 |
| Inactivity (hours/day) | 12.3  (12.0, 12.7) | 12.8  (12.4, 13.1) | 12.2  (11.9, 12.6) | 12.2  (11.9, 12.5) | 12.3  (12.0, 12.7) | 12.8  (12.3, 13.3) | 0.051 | 0.020 |
| Intensity of the most active continuous 30 minutes (mg) | 37.7  (27.6, 47.8) | 48.3  (39.1, 57.5) | 46.8  (37.3, 56.4) | 51.8  (43.2, 60.4) | 52.1  (42.0, 62.2) | 103.4  (89.6, 117.2) | <0.001 | <0.001 |
| Intensity of the most active continuous 10 minutes (m*g*) | 63.3  (50.1, 76.5) | 79.5  (67.4, 91.6) | 68.9  (56.4, 81.5) | 77.7  (66.4, 89.1) | 88.0  (74.8, 101.3) | 181.8  (163.7, 200.0) | <0.001 | <0.001 |
| **Sleep variables** |  |  |  |  |  |  |  |  |
| Total sleep time (hr/night) | 6.7  (6.5, 7.0) | 6.38  (6.17, 6.59) | 6.57  (6.37, 6.78) | 6.46  (6.27, 6.65) | 6.48  (6.3, 6.7) | 6.14  (5.84, 6.44) | 0.092 | 0.114 |
| Sleep efficiency (%) | 83.5  (82.3, 84.7) | 82.7  (81.6, 83.9) | 88.4  (87.3, 89.5) | 86.5  (85.5, 87.5) | 88.8  (87.6, 90.0) | 87.4  (85.6, 89.0) | <0.001 | 0.231 |
| Sleep midpoint variability (minutes) | 138.2  (124.7, 151.7) | 135.7  (122.9, 148.4) | 37.1  (24.4, 49.9) | 31.6  (20.0, 43.1) | 36.2  (22.7, 49.7) | 46.7  (28.3, 65.0) | <0.001 | 0.333 |

Data reported as marginal mean (95% CI). Adjusted for age, sex, ethnicity, deprivation, number of comorbidities, season of data collection, and number of wear days (physical activity variables) or wear nights (sleep variables).

M10 sessions p for group <0.001, group*sex 0.074

M30 sessions p for group < 0.001 group*sex 0.069

## Supplementary figures

**Supplementary Figure S1: Flow of data inclusion**

**Supplementary Figure S2: Odds ratios of not meeting 150 minutes of MVPA per week across acute illness severity.**


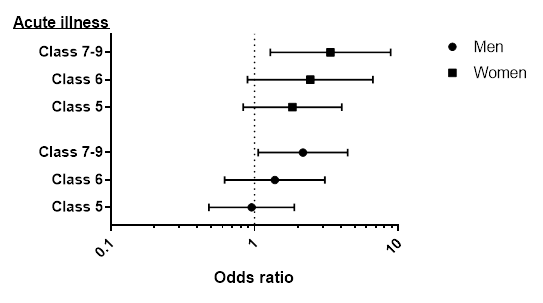


Data reported as odds ratios (95% CI). Reference group: Class 3-4. Analysis adjusted for age, sex, ethnicity, deprivation, number of comorbidities, season of data collection, and number of wear days. p for class =0.018, p for class x sex <0.001.

**Supplementary Figure S3: Odds ratios of not meeting 150 minutes of MVPA per week across recovery clusters.**


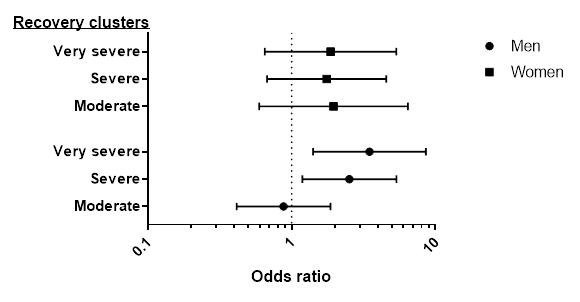


Data reported as odds ratios (95% CI). Reference group: Mild. Analysis adjusted for age, sex, ethnicity, deprivation, number of comorbidities, season of data collection, and number of wear days. p for class =0.012, p for cluster x sex =0.047.

Data display the proportion within each study achieving 0, 1, 2 and 3-7 days per week with a bout of 10 minutes (top panel) or 30 minutes (bottom) at least moderate-intensity physical activity. Analysis adjusted for age, sex, ethnicity, deprivation, number of comorbidities, season of data collection and number of wear days. 10-minute bouts: p for difference by studies = <0.001, p for difference by sex <0.001, p for sex x group = 0.074. 30-minute bouts: p for difference by studies = <0.001, p for difference by sex <0.001, p for sex x group = 0.069

**Supplementary Figure S4: proportion of participants within PHOSP, CODEC and SWL undertaking continuous bouts of physical activity.**


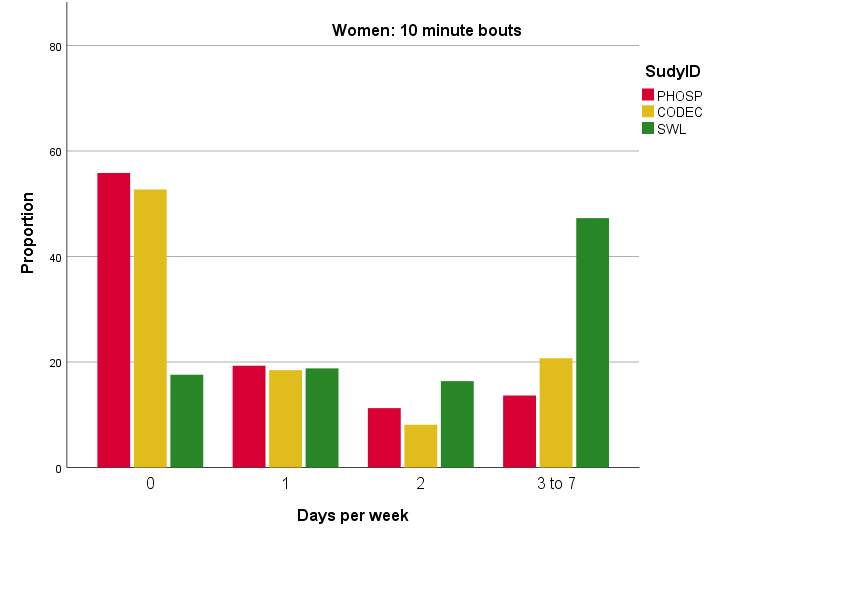

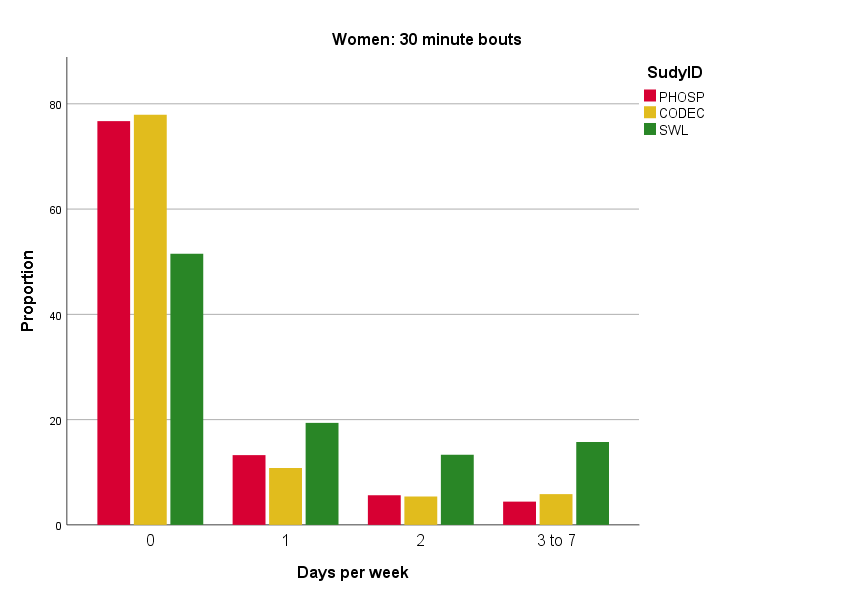

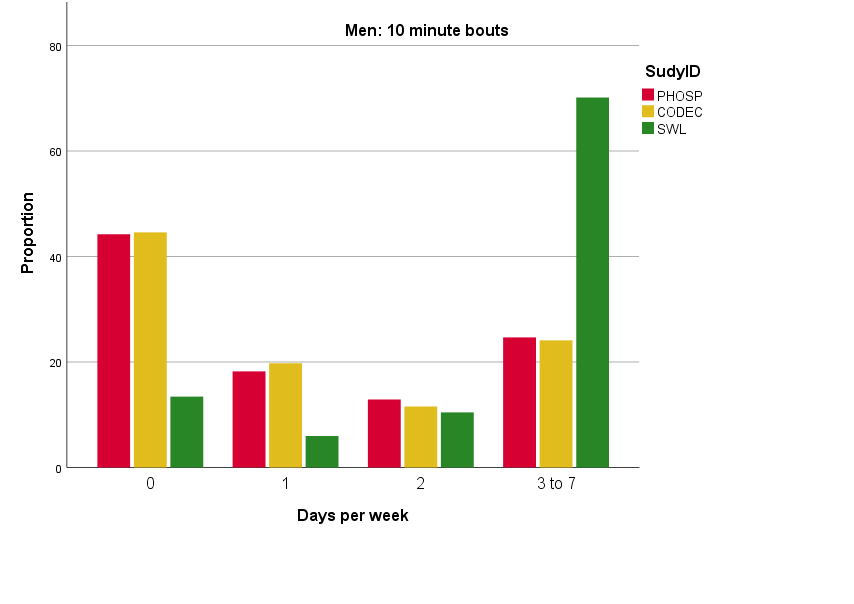

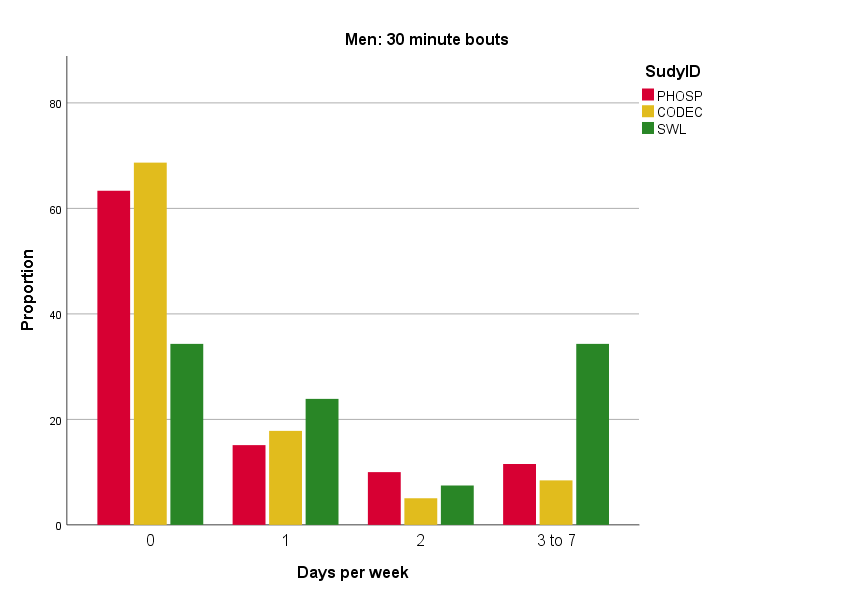


# References

1. Migueles JH, Rowlands AV, Huber F et al. GGIR: A research community-driven open-source R-package for generating physical activity and sleep outcomes from multi-day raw accelerometer data. *J Measure Phys Behav* 2019;2:188-196. doi.org/10.1123/jmpb.2018-0063

2. Van Hees V, Sabia S, Jones SE et al. Estimating sleep parameters using an accelerometer without sleep diary. *Scientific Reports* 2018;8:12975(2018). doi: 10.1038/s41598-018-31266-z

3. Jones SE, van Hees V, Mazzotti DR et al. Genetic studies of accelerometer-based sleep measures yield new insights into human sleep behaviour. *Nature Communications* 2019;10:1585. doi: 10.1038/s41467-019-09576-1

4. Wendt A, da Silva IC, Gonçalves H, Menezes A, Barros F, Wehrmeister FC. Short-term effect of physical activity on sleep health: a population-based study using accelerometry. Journal of Sport and Health Science 2020. doi: 10.1016/j.jshs.2020.04.007

5. Hildebrand M, van Hees VT, Hansen BH, Ekelund U. Age group comparability of raw accelerometer output from wrist- and hip-worn monitors. *Med Sci Sports Exerc* 2014;46:1816-24. DOI: 10.1249/mss.0000000000000289

6. Hildebrand M, Hansen BH, van Hees VT, Ekelund U. Evaluation of raw acceleration sedentary thresholds in children and adults*. Scand J Med Sci Sports* 2017;27:1814-23. /doi.org/10.1111/sms.12795

7. Troiano RP, Berrigan D, Dodd KW, et al. Physical activity in the United States measured by accelerometer. *Med Sci Sport Exerc* 2008;40:181-188. DOI: 10.1249/mss.0b013e31815a51b3

8. Marshall JC, Murthy S, Diaz J, et al. A minimal common outcome measure set for COVID-19 clinical research. *The Lancet Infectious Diseases* 2020;20(8):e192-7. doi.org/10.1016/S1473-3099(20)30483-7

9. Evans RA, McAuley H, Harrison EM, et al. Physical, cognitive, and mental health impacts of COVID-19 after hospitalisation (PHOSP-COVID): a UK multicentre, prospective cohort study. *The Lancet Respiratory Medicine* 2021;9(11):1275-87. doi.org/10.1016/S2213-2600(21)00383-0

10. Yorke J, Moosavi SH, Shuldham C, Jones PW. Quantification of dyspnoea using descriptors: development and initial testing of the Dyspnoea-12. *Thorax* 2010;65(1):21-6. doi: 10.1136/thx.2009.118521

11. Yellen SB, Cella DF, Webster K, et al. Measuring fatigue and other anemia-related symptoms with the Functional Assessment of Cancer Therapy (FACT) measurement system. *J Pain Symptom Manage* 1997;13(2): 63-74. doi: 10.1016/s0885-3924(96)00274-6

12. Johnson SU, Ulvenes PG, Øktedalen T, Hoffart A. Psychometric properties of the general anxiety disorder 7-item (GAD-7) scale in a heterogeneous psychiatric sample. *Front Psychol* 2019;10:1713. doi.org/10.3389/fpsyg.2019.01713

13. Levis B, Benedetti A, Thombs BD. Accuracy of Patient Health Questionnaire-9 (PHQ-9) for screening to detect major depression: individual participant data meta-analysis. *BMJ* 2019; 365:l1476. doi.org/10.1136/bmj.l1476

14. Weathers FW, Litz BT, Keane TM, et al. The PTSD Checklist for DSM-5 (PCL-5). 2013. [www.ptsd.va.gov](http://www.ptsd.va.gov).

15. Guralnik JM, Simonsick EM, Ferrucci L, et al. A short physical performance battery assessing lower extremity function: association with self-reported disability and prediction of mortality and nursing home admission*. J Gerontol* 1994;49(2):M85-M94. doi: 10.1093/geronj/49.2.m85.

16. Nasreddine ZS, Phillips NA, Bédirian V, et al. The Montreal Cognitive Assessment, MoCA: a brief screening tool for mild cognitive impairment. *J Am Geriatr Soc* 2005;53(4):695-9. doi: 10.1111/j.1532-5415.2005.53221.x

17. Edwardson CL, Biddle SJ, Clarke-Cornwell A et al. A three-arm cluster randomised controlled trial to test the effectiveness and cost-effectiveness of the SMART Work & Life intervention for reducing daily sitting time in office workers: study protocol. *BMC Public Health* 2018;18(1):1-3. https://doi.org/10.1186/s12889-018-6017-1

18. Rowlands AV, Plekhanova T, Yates T, et al. Providing a basis for harmonization of accelerometer-assessed physical activity outcomes across epidemiological datasets. *J Measure Phys Behav* 2019;2: 131-142. doi.org/10.1123/jmpb.2018-0073.

19. Plekhanova T, Rowlands AV, Yates T, et al. Equivalency of sleep estimates: comparison of three research-grade accelerometers. *J Measure Phys Behav* 2020;3,294-303. doi: 10.1123/jmpb.2019-0047.

20. Brady EM, Hall AP, Baldry E, et al. Rationale and design of a cross-sectional study to investigate and describe the chronotype of patients with type 2 diabetes and the effect on glycaemic control: the CODEC study. *BMJ Open* 2019;9:e027773. doi: 10.1136/bmjopen-2018-027773

21. Henson J, Rowlands AV, Edwardson CL, et al. Physical behaviours and chronotype in people with Type 2 diabetes. *BMJ Open Diabetes Research and Care* 2020;8:e001375. doi: 10.1136/bmjdrc-2020-001375
